# Synthetic Fertilizer Increases Denitrifier Abundance and Depletes Subsoil Total N in a Long-Term Fertilization Experiment

**DOI:** 10.3389/fmicb.2020.02026

**Published:** 2020-08-31

**Authors:** Ying Wang, Hongfei Ji, Rui Wang, Yaxian Hu, Shengli Guo

**Affiliations:** ^1^Key Laboratory of Soil Erosion and Dryland Farming on the Loess Plateau, Northwest A&F University, Yangling, China; ^2^Institute of Soil and Water Conservation, Chinese Academy of Sciences and Ministry of Water Resources, Yangling, China

**Keywords:** denitrifier, nitrifier, gene abundance, subsoil, Chinese Loess Plateau

## Abstract

Chronic synthetic nitrogen (N) application can result in a significant accumulation of nitrate in the subsoil, which could alter subsoil N cycle and subsequently affect subsoil N levels. To understand how elemental interactions affect the cycle and storage of subsoil N, we examined the soils receiving no fertilizer control (CK), 30-year applications of synthetic fertilizer (CF), and CF plus organic manure (CF + OM). The N cycling microbial groups and activity were investigated through analyzing abundance of bacteria, nitrifiers and denitrifiers, potential nitrification (PNA) and denitrification (DEA) rates in the topsoil (0–20 cm) and subsoil depths (20–80 cm). Compared with the CK, the CF application increased subsoil nitrate but reduced or did not change subsoil microbial biomass N and total N. Corresponding to the increased nitrate, the abundances of denitrifiers increased in the CF subsoils. By contrast, the abundances of nitrifiers increased in the CF topsoil. Significant correlation between the abundances of nitrifiers and soil PNA was found in the topsoil, while significant correlation was also found in the subsoil between the abundances of *nirS*- and/or *nirK*-type denitrifiers and DEA. These results suggest that the depleted or less changed subsoil total N by CF application might be partly related to the enriched denitrifiers groups and the related potential activity. The contrasting responses of nitrifiers and denitrifiers in the CF subsoil indicate a decoupling of both processes. Our findings highlight that the leached nitrate by synthetic fertilizer addition not only occurs as an environmental risk causing groundwater contamination but may also alter the subsoil N cycle through the denitrifier groups.

## Introduction

Nitrate leaching and accumulation in soil profiles have been found in crop lands worldwide (Ju et al., 2004; Stumborg et al., 2007; López-Bellido et al., 2013), which is mainly caused by the chronic application of synthetic nitrogen (N) fertilizer in improving crop yield. The excessive accumulated nitrate in soil profile can contribute to many environmental problems, e.g., NO_x_ emissions (Galloway et al., 2008; Schmidt et al., 2011), eutrophication and ground water contamination (Tilman et al., 2001; Galloway et al., 2008). Moreover, the increased nitrate in subsoil profile is likely to affect soil N pools and cycling rates (Treseder, 2008; Lu et al., 2011; Jian et al., 2016), which might also alter soil carbon (C) pools and cycling. Therefore, such impacts are important for the regulation of soil fertility in the soil profile (Schmidt et al., 2011; Shang et al., 2014; Jian et al., 2016). N fertilization has been shown to increase soil organic C content in both top- and subsoils (Frey et al., 2014; Li et al., 2014) by the positive effects of the fertilizer on crop growth and, in turn, crop C return (Purakayastha et al., 2008), or through reducing soil respiration (Frey et al., 2014; Chen et al., 2019). In contrast, total N in the subsoil was depleted or less changed by fertilization (Ju et al., 2004; Li et al., 2014). The direction and extent of shift in soil N cycling could occur via two fundamental ways. Firstly, fertilization increases soil N availability and subsequently crop C return (Hodgkinson et al., 2017; Hirte et al., 2018), which might improve soil N retention. Secondly, fertilization changes the rate of microbial transformation of N which controls retention (Mooshammer et al., 2014) and loss of N from soil (Kuypers et al., 2018). Therefore, the depleted or less changed subsoil total N by fertilization may be more tightly coupled to microbial processes than to N input *per se*.

Nitrogen losses by nitrate leaching and gaseous N emissions are caused by both nitrifiers and denitrifiers. Several studies have reported the effect of synthetic fertilization on nitrification and nitrifier groups [ammonia-oxidizing archaea (AOA) and ammonia-oxidizing bacteria (AOB)] in the soil profile (Leininger et al., 2006; Wang et al., 2014). However, how fertilization and the accumulated nitrate in the soil profile affect the distributions of denitrifiers and the related denitrification in the subsoil profile remains largely unexplored. For instance, AOB abundance in soil profile was increased by N addition (Wang et al., 2014) and related to soil nitrification rate (Di et al., 2010), while AOA abundance was less affected (Leininger et al., 2006; Wang et al., 2014). The abundance of denitrifier groups in topsoil has been shown to increase after N addition (Sun et al., 2015; Yin et al., 2015). However, how denitrifier groups and denitrification in subsoil respond to fertilization is barely studied. Furthermore, organic manure amendment has been shown to enhance denitrification activity and the related N_2_/N_2_O ratios in topsoil (Kramer et al., 2006), and increase the relative abundance of nitrifiers (calculated from 16S rRNA pyrosequencing results) in subsoil compared to synthetic N application (Li et al., 2014). Such changes may exert critical influences on top- and subsoil N cycling. Therefore, a systematic investigation is required to assess the possible impacts of fertilizer regimes and the resulted nitrate accumulation on the subsoil N cycling and the related microbial communities.

Thus, we investigated soil N pools, potential soil microbial respiration, nitrification and denitrification rates, total bacteria, nitrifiers and denitrifiers in the soil profile (topsoil: 0–20 cm; subsoil: 20–40, 40–60, and 60–80 cm) in a 30-year field experiment on the Loess Plateau of China. Two typical fertilizer treatments (synthetic fertilizer alone, CF and CF plus organic manure, CF + OM) were selected to compare with the unfertilized treatment. Our preceding research found that CF and CF + OM increased the ratios of nitrifiers to nitrate reducers in topsoil (at March), suggesting a loss of N via leaching (Wang et al., 2019). Earlier studies on this site also showed significant nitrate and organic C accumulations in the soil profile after long-term fertilization (Guo S. et al., 2010). In the current study, the objectives were to explore the responses of subsoil microbial communities and activity to CF and CF + OM applications and their links. We hypothesized that (i) the increases of nitrifiers and denitrifiers in the soil profile will differentiate in the CF treatment and (ii) the enriched denitrifiers in the CF subsoil will link to soil nitrate content and denitrification, which may affect soil total N.

## Materials and Methods

### Experimental Design and Soil Sampling

The experiment was conducted in the Changwu Agro-ecological Experimental Station on the Loess Plateau 107°40′E, 35°12′N, altitude 1220 m, Shaanxi Province, China. This site has a semi-arid climate and represents a typical rain-fed agricultural area with annual mean precipitation 542 mm (1996–2005). The soil in this study area is uniform loam (Cumulic Haplustolls; USDA Soil Classification System) developed from loess deposits. Soil pH is about 8.5 and barely affected by fertilizer applications (Guo J. H. et al., 2010).

The long-term fertilizer field experiment was started in 1984 with a continuous winter wheat (*Triticum aestivum* L.) grown system, which was described in detail in our previous reports (Guo S. et al., 2010). In brief, the fertilization experiment included no fertilizer (CK), synthetic N fertilizer (N), organic farmyard manure (OM), phosphorus (P), P plus OM, N and P fertilizers (CF), N plus OM, and CF plus OM (CF + OM). The following two treatments, as common local fertilization regimes, were selected to compare with no fertilizer application (CK): synthetic fertilizers N and P (CF), and CF plus organic manure (CF + OM). The fertilizer N and P were applied in the form of urea (120 kg N ha^–1^ per year) and superphosphate (40 kg P_2_O_5_ ha^–1^ per year) (Table 1). Organic farmyard manure (10.7 and 1.16 g kg^–1^ as total C and N, respectively) was applied at a rate equivalent to 87 kg N ha^–1^ per year. Each treatment had three replicates, and the plot size was 10.3 × 6.5 m^2^, separated by 0.5-m buffer strips. All fertilizers were applied in September, 5–7 days prior to wheat sowing. No irrigation was applied.

**TABLE 1:** Fertilizer application rates and soil sample collection in each treatment.

**Treatment**	**N application rate (kg N ha^–1^ yr^–1^)**	**P application rate (kg P_2_O_5_ ha^–1^ yr^–1^)**	**M application rate (ton ha^–1^ yr^–1^)**	**Soil sample**
CK	0	0	0	Three 0–80 cm vertical soil cores in each field plot were collected and divided into four sections (0–20, 20–40, 40–60, and 60–80 cm).
CF	120	40	0	
CF + OM	120	40	75	

The soil samples of this study were collected in March 2014. Two soil cores (4 cm inner diameter) were taken from each field plot, in order to minimize the damage to the plot. For each core, soil subsamples from four layers, 0–20 cm, 20–40 cm, 40–60 cm, and 60–80 cm, were collected to form an 80-cm-long vertical profile. The 0–20 cm soil was defined as topsoil, and the other depths in the soil profile as subsoil. Samples taken at the same depth layer in each plot were then mixed to form one composite sample, i.e., three replicates for each treatment. In total, 36 soil samples were obtained (3 treatments × 4 depths × 3 replicates). The visible plant roots, stones, and debris were manually removed. All samples were passed through a 2.0-mm sieve, and each was then divided into three subsamples. One subsample was stored at −80°C for DNA extraction, one at 4°C for microbiological analysis (within 3 weeks), and the third subsample was air-dried for soil physicochemical analysis. Soil biochemical parameters were quantified as our previous study (Wang et al., 2017), including soil total organic C (SOC), total N (TN), ammonium, nitrate, available P (AP), dissolved organic C (DOC), and microbial biomass C and N (MBC and MBN).

### Soil Microbial Activity

Soil microbial respiration was measured according to the method modified from Enwall et al. (2007). In brief, 10 g fresh soil from each replicate was placed in a 125 mL glass bottle, sealed with a rubber plug, and then incubated at 25°C for 72 h. At the end of the incubation, headspace CO_2_ concentrations were measured via gas chromatography (HP7890B, Agilent Technologies, CA, United States).

Potential nitrification activity (PNA) was measured by the shaken soil slurry method as described in our previous study (Wang et al., 2012). Briefly, 5.0 g fresh soil per sample was added to a 50 mL centrifuge tube containing 20 mL of phosphate buffer solution (g L^–1^: KCl, 0.2; NaCl, 8.0; Na_2_HPO_4_, 0.2; NaH_2_PO_4_, 0.2; pH 7.5) with 1 mM (NH_4_)_2_SO_4_. The suspension was incubated on an orbital shaker at 25°C for 24 h. Nitrate concentration was determined with an automated flow injection analyzer (FLOWSYS, Italy) after extraction by 5 mL of 2 M KCl.

Potential denitrification activity (DEA) was measured as described in our previous study (Wang et al., 2019). In brief, 10 g soil per sample was weighted into a 100 ml glass bottle containing 10 ml sterile distilled water. Denitrifying conditions were induced by flushing the headspace with pure nitrogen gas (99.9%) for 10 min. After the bottles were re-adjusted to atmospheric pressure, 15 mL headspace gas was substituted with pure acetylene (99.9%). Bottles were shaken on an orbital shaker for 30 min and then incubated at 25°C for 12 h. Gas samples (15 mL) were taken and analyzed for N_2_O by a gas chromatograph (HP7890B, Agilent Technologies, CA, United States).

### Soil DNA Extraction and Quantitative Real-Time PCR

Total soil DNA was extracted from 0.5 g soil by FastDNA^®^ Spin Kit for Soil (MP Biomedicals, Cleveland, OH, United States) according to the manufacturer’s instructions. The purified DNA was diluted with 100 μL sterilized water and checked for quality and quantity using a NanoDrop Spectrophotometer.

Quantitative analysis was conducted for fragments of the bacterial 16S rRNA, archaeal 16S rRNA, and six functional genes related to the N cycle. The N-cycle-related functional genes included bacterial and archaeal *amoA* (coding for the ammonia monooxygenase; AOB and AOA; responsible for ammonia oxidization in nitrification), *nirS* and *nirK* (both coding for a nitrite reductase; nitrite reducers), *nosZ* (coding the N_2_O reductase; nitrous oxide reducers) and *narG* (coding for the nitrate reductase; nitrate reducers). Quantitative real-time PCR (qPCR) was performed in a CFX96 Real-Time system (Bio-Rad) via a three step thermal cycling procedure as described in our previous study (Wang et al., 2019). It should be caution when comparing 16S rRNA gene copies between treatments, since the 16S rRNA gene copies per cell vary between 1 and 15 (Hallin et al., 2009). Duplicate technical replicates were performed for each sample. The *R*^2^ value for each standard curve exceeded 0.99, which indicated linear relationships across the concentration ranges used in this study. A dissociation step was added at the end of the real-time PCR to assess amplification quality. The specificity was further evaluated by running 30 randomly selected PCR products (for each gene) on a 1.5% (w/v) agarose gel.

### Statistical Analysis

The relative changes of soil variables (C and N pools and microbial activity) were compared between the CF and the unfertilized soils and the CF + OM and CF soils, to reflect the different responses across soil depths in the two fertilization regimes.

Repeated measure analysis was used to test the significance of treatments (CK, CF, and F + OM) across depths on soil parameters and microbial abundance; *post hoc* analyses (where appropriate) were performed using Bonferroni’s multiple comparison test at *P* < 0.05. One-way analysis was used to test the significance of treatments (CK, CF, and F + OM) on the abundance of microbial communities in each soil depth. Normal distribution of the data was tested by Q–Q plot. If necessary, data were log transformed to achieve a normal distribution. Possible changes in microbial abundances along soil depth in each of the three fertilization treatments were tested by linear regressions between soil depth and microbial abundances. Spearman’s correlation coefficients were used to test the relationships between microbial abundances, microbial activities and soil properties. The statistical analyses were performed using SPSS 20.0 software (SPSS Inc., Chicago, United States).

Significant spearman’s correlation coefficients between all pairs of N-cycling microbial groups were considered as an indication of similar ecological requirements and microhabitats between these groups. Correlation network mapping, mainly by visually representing nodes (microbial groups) connected by edges (level of the correlation observed between abundances), was generated using the Fruchterman-Reingold in the open source Gephi software (Bastian et al., 2009). To examine the effects of fertilization regime on the correlation network across soil depths, the maps were built for data obtained from the three fertilizer regimes in each soil depth.

## Results

### Soil C and N Pools and Microbial Activity

**FIGURE 1:**
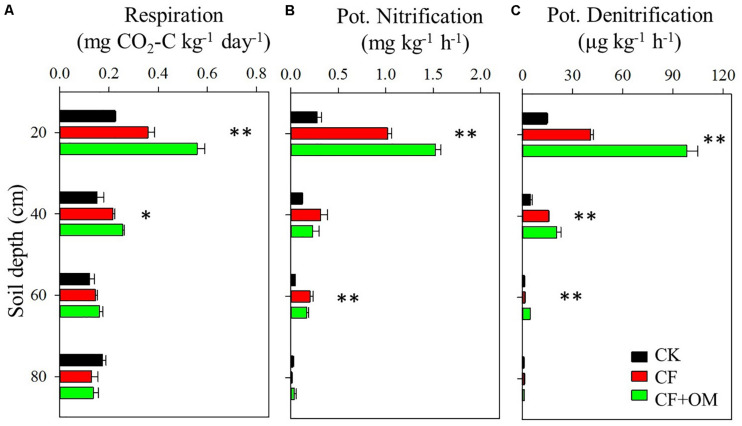
Soil microbial activities in the soil profile (0–80 cm depths) under long-term fertilizer treatments. **(A)** Potential soil microbial respiration, **(B)** potential nitrification activity, and **(C)** potential denitrification activity. ^∗^*P* < 0.05; ^∗∗^*P* < 0.01. Error bars are standard errors (*n* = 3). CK: unfertilized soil; CF: synthetic fertilizer; CF + OM: CF plus organic manure.

**FIGURE 2:**
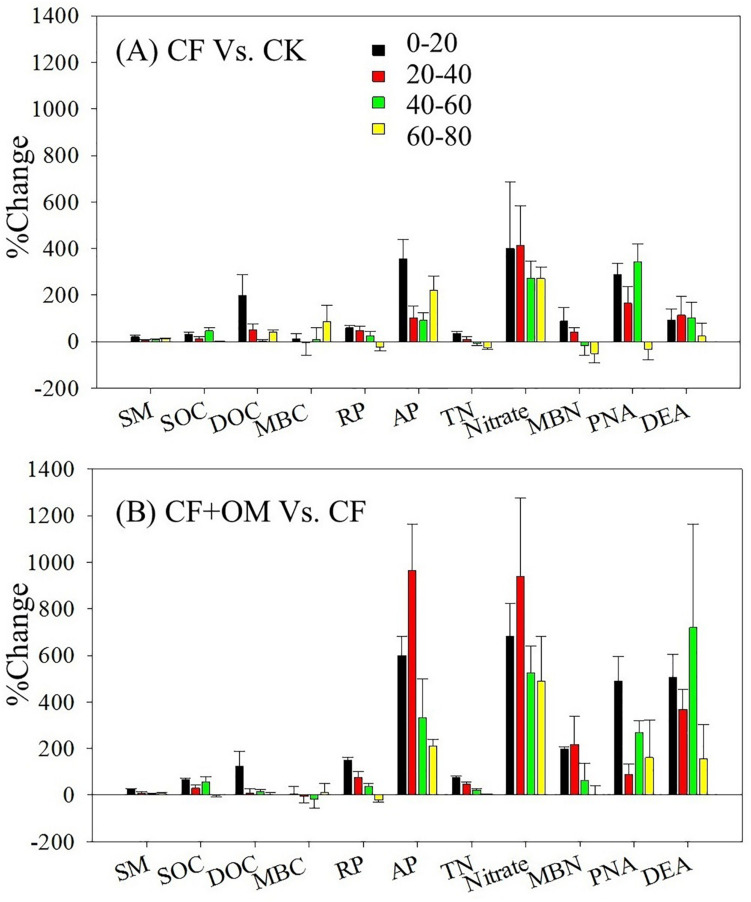
Percent change of soil parameters and microbial activity under different treatments after 30 years of fertilizer applications. SM, soil moisture; SOC, soil organic C; DOC, dissolved organic C; MBC, microbial biomass C; RP, soil microbial respiration; AP: available P; TN: total N; MBN, microbial biomass N; PNA: potential nitrification rate; DEA: potential denitrification rate. Error bar is standard error of the means (*n* = 3). CK: unfertilized soil; CF: synthetic fertilizer; CF + OM: CF plus organic manure.

Compared to the unfertilized treatment (CK), the two fertilizer treatments generally increased soil organic C (SOC), total N, dissolved organic C, microbial biomass N, nitrate, and available P in the soil profile, except total N and MBN in the CF treatment in the 40–80 cm depths (Supplementary Figure S1). The two fertilizer treatments generally resulted in a significant increase in soil microbial respiration, potential nitrification (PNA), and denitrification (DEA) in the 0–60 cm depths, with a greater increase in the CF + OM than the CF (Figure 1). More specifically, soil microbial respiration in the CF treatment in the 0–60 cm depths was 59%, 48%, and 25% higher than that in the CK treatment, respectively, while the increases in the CF + OM treatment were 149%, 76%, and 36%, respectively. PNA in the 0–60 cm depths was increased by 287%, 164%, and 342% (CF), and 490%, 89%, and 266% (CF + OM), respectively. DEA in the 0–60 cm depths was increased by 92%, 114%, and 103% (CF), and 507%, 367%, and 719% (CF + OM), respectively. The percent change analysis revealed that the rate of change induced by CF and CF + OM was generally greater for soil available P, nitrate, MBN, PNA, and DEA than for TOC, DOC, MBC, and soil microbial respiration (Figure 2). Compared to the CF treatment, the CF + OM treatment generally induced a higher rate of change in soil available P, nitrate, MBN, and DEA.

### Abundances of Prokaryotes and N-Cycling Microbial Groups

**FIGURE 3:**
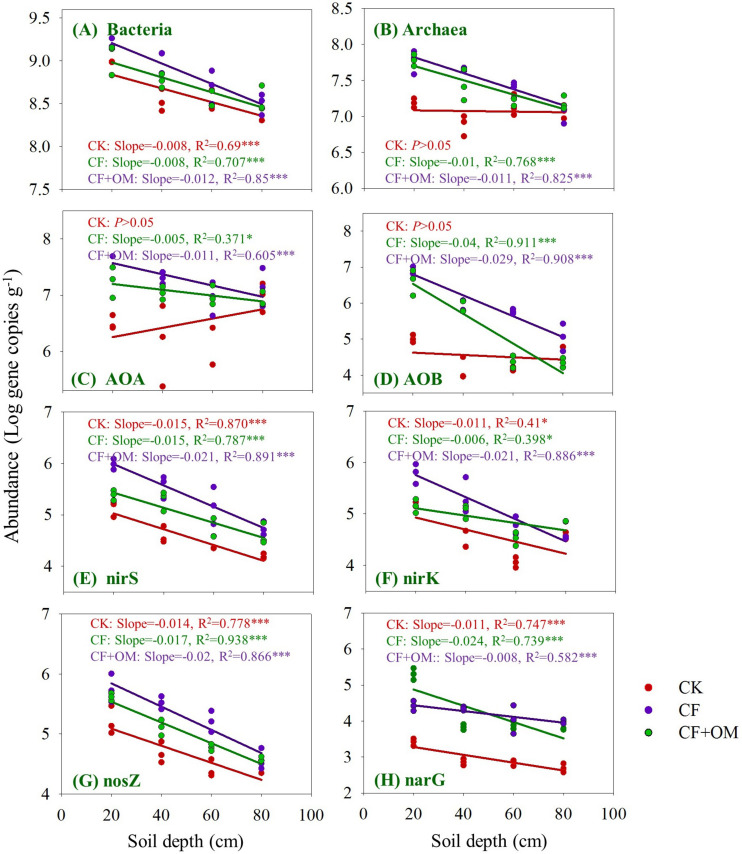
Changes in the abundances of prokaryotes, nitrifying and denitrifying microbial groups in the soil profile (0–80 cm depths) under different treatments after 30 years of fertilizer applications. **(A)** Bacteria, **(B)** archaea, **(C)** AOB, **(D)** AOA, **(E)** *nirS*, **(F)** *nirK*, **(G)** *nosZ*, and **(H)** *narG* genes. The lines denote the linear regressions across soil depth. ^∗^*P* < 0.05; ^∗∗∗^*P* < 0.001. The abundance of each gene was expressed as the log gene copy numbers g^–1^ dry soil. CK: unfertilized soil; CF: synthetic fertilizer; CF + OM: CF plus organic manure.

The bacterial abundance quantified by 16S rRNA gene copy numbers linearly decreased from 8.7 × 10^8^ to 2.6 × 10^8^ per g dry soil with soil depth in the CK treatment, while archaeal 16S rRNA gene copy numbers showed no difference between soil depths (Figures 3A,B). Compared to the CK treatment, bacterial abundance in the 20–40 cm depth was increased by the CF + OM by 200%; archaeal abundance was increased by the CF in the 0–40 cm depths (268–295%) and by the CF + OM in the soil profile (0–60 cm; 88–471%).

The abundances of AOA and AOB in the CK soil profile ranged from 1.0 × 10^7^ to 2.8 × 10^6^ copies per g dry soil and from 1.0 × 10^5^ to 1.4 × 10^4^ copies per g dry soil, respectively, and were not changed between soil depths (Figures 3C,D). The AOA and AOB abundances in the two fertilizer treatments were higher than that in the CK treatment and decreased with soil depth. Specifically, the abundances of AOB and AOA increased in the CF in the 0–40 cm depths by 49–68 folds and 5–20 folds, respectively; the increases in the CF + OM were 85, 76, and 38 folds (0–60 cm depths), and14 and 31 folds (0–40 cm depths), respectively. In addition, AOB abundance in the CF + OM in the 40–80 cm depths was 26 and 6.5 folds higher than that in the CF, respectively.

The abundance of denitrifier groups in the soil profile linearly decreased with soil depth and generally increased with the CF and CF + OM applications (Figures 3E–H). Specifically, the abundance of *nirS*-type nitrite reducers in the subsoil was 1.4–4 folds higher in the CF than in the CK; the increases in the CF + OM soil profile were 5.9 (topsoil) and 2.6–8.6 folds (subsoil), respectively. Similar increases were also observed for the *nirK*-type nitrite reducers in the CF and CF + OM; while the increased abundance of *nosZ*-nitrous oxide reducers occurred in the 0–40 cm (CF) and 0–60 cm (CF + OM) depths, respectively. The abundance of *narG*-nitrate reducers in the soil profile was increased by 8.4–78.8 folds (CF) and 9.2–29.5 folds (CF + OM), respectively. In addition, compared to the CF treatment, the CF + OM treatment increased *nosZ* and *narG* abundances in the subsoil, but decreased *nirK* (60–80 cm) and *narG* abundances (0–20 cm). Generally, the soil in 20–40 cm was the most responsive depth for nitrifiers and denitrifiers to fertilizer applications.

The vertical spatial variation in each of the N-cycling microbial groups down through the soil profile was compared between the fertilizer treatments (Figure 3). Through the soil profile, the vertical spatial decay relationship slopes of the bacteria and all the N-cycling microbial groups were generally steepest in the CF + OM treatment. The slope in the CF treatment was higher for archaea, AOB, *nosZ*, and *narG* than that in the CK treatment, but remained similar for bacteria and *nirS*-type nitrite reducers. The abundance of bacteria, *nirS*, *nirK*, *nosZ*, and *narG* in the CK was linearly decreased through the soil profile, while that of archaea, AOA, and AOB was not changed. The vertical spatial decay relationship slope of the ratios of functional gene to 16S rRNA gene (bacteria + archaea) showed similar trend as each of the functional genes (Supplementary Figure S2). This suggests that the changes of N-cycling microbial groups in the studied soil profiles were generally not affected by the variability in the soil microbial biomass.

**FIGURE 4:**
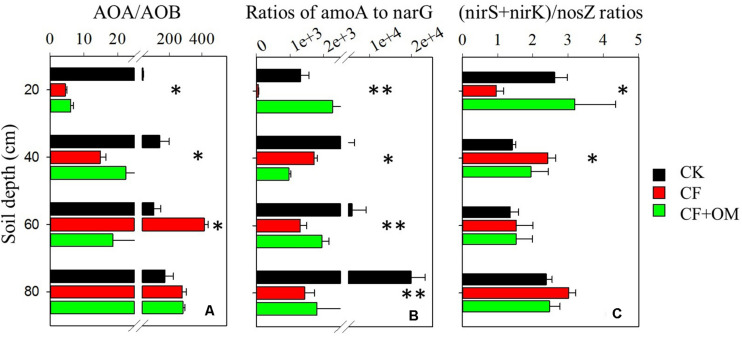
Effects of long-term fertilizer applications on ratios of **(A)** AOA/AOB, **(B)** *amoA*/*narG*, and **(C)** (*nirK* + *nirS*)/*nosZ*. ^∗^*P* < 0.05; ^∗∗^*P* < 0.01. Error bars are standard errors (*n* = 3). CK: unfertilized soil; CF: synthetic fertilizer; CF + OM: CF plus organic manure.

The ratios of abundances of *amoA* gene (AOA + AOB) to *narG* gene and that of abundances of the sum of *nirS* and *nirK* genes to *nosZ* gene were used as indicators of nitrate leaching and denitrification-derived gaseous N loss potentials, respectively. In the topsoil, CF application decreased the *amoA* to *narG* and (*nirS* + *nirK*)/*nosZ* ratios, compared to the CK treatment (Figures 4B,C). In the subsoil depths, the *amoA* to *narG* ratios were substantially reduced by the CF and CF + OM treatments. In contrast, the CF and CF + OM treatments increased the (*nirS* + *nirK*)/*nosZ* ratios in the 20–40 cm depth but did not affect them in the 40–80 cm depths.

### Correlations Among Soil Microbial Activity and Abundances of N-Cycling Microbial Groups

**FIGURE 5:**
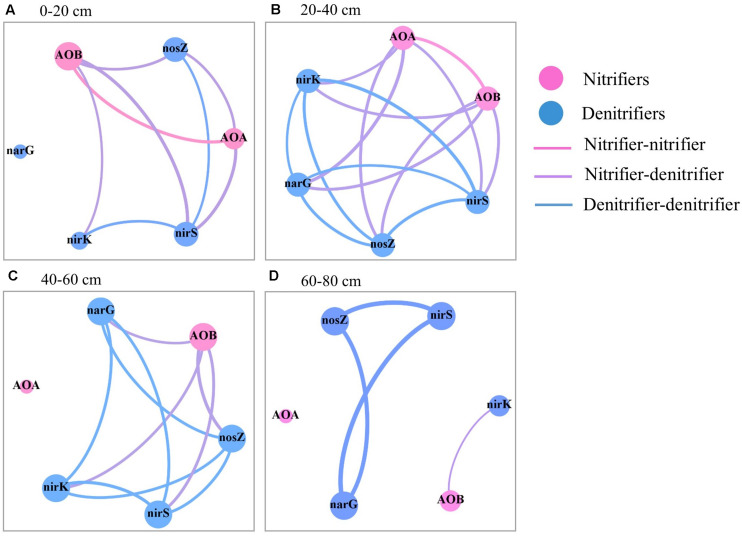
Spearman’s correlation between the abundances of the six N-cycling groups in the soil profile (0–80 cm depths) after 30 years of fertilizer applications. **(A)** 0–20 cm, **(B)** 20–40 cm, **(C)** 40–60 cm, and **(D)** 60–80 cm. The size of each node is proportional to the number of connections. The link thickness was proportional to correlation coefficient.

In the topsoil, there were strong correlations for the abundances of AOB, N_2_O reducers, and *nirK-* and *nirS*-type nitrite reducers (Figure 5 and Supplementary Table S1). While in the subsoil (20–60 cm), there were strong correlations between the abundances of all N-cycling microbial groups except AOA in 40–60 cm. Strong correlations were observed for the abundances of nitrate reducers, N_2_O reducers, and *nirS*- nitrite reducers in the 60–80 cm depth.

In the soil profile, the AOB abundance and the ratios of AOA to AOB abundances (AOA/AOB) were positively correlated with soil PNA; the abundances of *nirK-* and *nirS*-type nitrite reducers were correlated with DEA (Table 2). The significance of the correlation was changed when the correlation analysis was divided by soil depth. Specifically, significant correlation between the abundances of AOB and AOA and soil PNA was only found in the topsoil. In addition to the topsoil, significant correlations between the abundances of *nirS*- and/or *nirK*- type nitrite reducers and DEA were also found in the subsoils (20–60 cm).

**TABLE 2:** Spearman’s correlation of soil potential nitrification (PNA) and denitrification activity (DEA) with the abundances of nitrifiers and denitrifiers.

**Soil depth (cm)**	**AOA**	**AOB**	**AOA/AOB**	***nirK***	***nirS***	***nosZ***	***narG***
**PNA**							
All	0.318	0.681**	0.715**	0.669**	0.790**	0.851**	0.574**
0–20	0.915**	0.733*	0.150	0.650	0.867**	0.683*	0.467
20–40	0.367	0.433	0.633	0.583	0.650	0.633	0.417
40–60	0.350	0.450	–0.017	0.467	0.483	0.667*	0.700*
60–80	–0.133	0.517	0.433	–0.333	0.100	–0.333	0.217
**DEA**							
All	0.405*	0.767**	0.793**	0.767**	0.843**	0.866**	0.588**
0–20	0.915**	0.883**	–0.050	0.550	0.917**	0.683*	0.417
20–40	0.817**	0.850**	0.717*	0.683*	0.683*	0.833**	0.833**
40–60	0.067	0.667*	0.717*	0.600	0.733*	0.600	0.300
60–80	0.300	0.233	0.033	0.083	0.217	0.117	0.367

* *P* < 0.05; ** *P* < 0.01.

## Discussion

### Responses of N Pools and Cycling in the Soil Profile to Fertilization

Our results showed that N cycling rates (PNA and DEA) in the subsoil were more responsive to fertilizer application than C-cycle-related variables (Figure 2). The greater response of N than C cycle variables has been observed in recent studies of the effect of environmental conditions (e.g., N addition, temperature, and moisture) on biogeochemical cycles (Rodríguez et al., 2014; Morillas et al., 2015; Durán et al., 2017). Such change might be explained in part by that N-cycle-related microbial groups are less diverse and redundant than that used soil organic C (Kowalchuk and Stephen, 2001; Philippot and Hallin, 2005). This was supported by the more responsive of the N-cycle-related microbial groups to fertilization regimes than the total bacteria in the current study (Figure 3). The enhanced subsoil N cycle in the CF might contribute to the depleted or less changed total N, since the CF had lower C input (such as crop root) and other nutrients in subsoil which may limit N retention. This is partly supported by that soil microbial decomposers tend to release an increasing fraction of organic N when their metabolic control switches from N to C limitation (Mooshammer et al., 2014). These results suggest that the studies focusing on the topsoil likely lead to limited understanding of how deeper soil depths function (Harrison et al., 2003). This indicates that failure to consider the deeper soil depths in fertilization research can result in an incomplete perspective and overly conservative assessment of fertilization effects on soil functioning. It is important to understand that we did not assess whether manure served as inoculum for introducing C and N cycling-related microbial taxa to the soil, or whether manure mainly served as substrate for soil C and N cycling processes. As a next step, it would be interesting to evaluate how microbial communities, C and N cycling processes in soils that have been unfertilized or fertilized with synthetic fertilizer for a long time would respond to manure additions over an extended period of time. It also remains to determine the amount and type of N taken up by wheat roots at different soil depth.

### Variations of Soil N-Cycling Microbial Groups in the CF and CF + OM Fertilizations

The CF application increased nitrifiers in the topsoil but increased denitrifiers in the subsoil, suggesting a differed distribution of nitrifiers and denitrifier groups in the CF vertical soil profile. The responses of nitrifiers to CF were partly consistent with previous findings on topsoil where AOB increased abundance in synthetic fertilized soils while AOA was less affected (Sun et al., 2015; Yin et al., 2015; Tang et al., 2016) or less responsive to N application than AOB (Carey et al., 2016). The differed responses of nitrifiers and denitrifiers in the CF might partly be due to the bioavailability of nitrogen and aerobic soil conditions within soil profile. Ammonium mainly derives from fertilizer application and presents in the upper soil profile, while nitrate is easy to move by leaching and then may enhance denitrifier growth in subsoil depths. This is supported by that significant correlation between the abundances of AOB and AOA and soil PNA was only found in the 0–20 cm depth while that of *nirS*- and/or *nirK*-type nitrite reducers and DEA was also found in the 20–60 cm depths (Table 2). The CF application increased the abundance of *nosZ* gene (0–40 cm depths) coding for nitrous oxide reductase which reduces N_2_O to N_2_. This indicates that the ratio of N_2_ to N_2_O as end product of denitrification is likely to be increased in the CF application soil. Therefore, these results in combination with the high concentration of nitrate in the fertilized soil profile in this and our previous studies (Guo S. et al., 2010) suggest that gaseous N losses are likely to be substantially increased in the chronic synthetic fertilized soil profile.

Additional organic manure in the CF + OM treatment resulted in a greater increase of nitrifier abundances in the soil profile and *nosZ* and *narG* abundances in the subsoil. The increased nitrifier abundances in the CF + OM treatment may partly explain previous findings that soil nitrification activity in deeper soil depths was increased by organic manure application (Khalili and Nourbakhsh, 2012; Wang et al., 2014). The enhanced abundance of *nosZ* gene in subsoil agrees with previous findings (Kramer et al., 2006; Barrett et al., 2016). The mechanism of the effect of additional organic manure on the subsoil nitrifiers and denitrifiers might be due to the increased bioavailability of organic C and diverse nutrients in subsoil (Khalili and Nourbakhsh, 2012). The *narG* in topsoil was lower in abundance in the CF + OM than in the CF, which might be due to the effect of C input by organic manure (Kramer et al., 2006). Organic manure addition has been shown to stimulate ammonium immobilization rate and may prevent nitrate buildup in topsoil (Wang et al., 2015), resulting in the limited growth of nitrate reducers. Further investigation is needed to explore the mechanism of synthetic effects of the CF and additional organic manure application on the distribution of N-cycling microorganisms and their ecological role in N immobilization in the field.

The soil in 20–40 cm was generally the most responsive depth for nitrifiers and denitrifiers to fertilizer applications. This was not consistent with the changes in soil parameters by fertilizer applications which was highest in topsoil (Li et al., 2014; Qiu et al., 2016). Such discrepancy might be explained by the following two mechanisms. First, the larger and more diverse microbial communities in topsoil may be less responsive to the fertilization-related environmental variation, due to the presence of functionally redundant microbial species (Saleem et al., 2016). The results in the current study and other reports showed that the abundance and biomass of microorganisms decreased with soil depth (Wang et al., 2014; Loeppmann et al., 2016). Second, in the topsoil, the strong control exerted by winter wheat might have dampened the effects of fertilizer application, whereas in the deeper depths the controls over N-cycling microorganisms might have been more dominated, and therefore more affected, by fertilizer-induced changes in nutrients and C availability (Loeppmann et al., 2016).

### Implications of Variations of Soil Parameters and N-Cycling Microbial Groups in the Soil Profile

Soil nitrate and available P were correlated with most of the nitrifying and denitrifying groups in the soil profile and across soil depths (Supplementary Table S2), suggesting their important roles in the distribution of N-cycling microbial groups and the associated soil N cycle. This is consistent with previous reports on topsoil (Sun et al., 2015; Tang et al., 2016). However, strong correlations between the abundances of N-cycling microbial groups were limited to part of groups in the 0–20 cm and 60–80 cm depths but found for all groups (except AOA in 40–60 cm) in the 20–60 cm depths (Figure 5). This suggests that the distributions of N-cycling microbial groups in the soil profile might be also affected by other factors such as substrate limitation and/or competition by winter wheat.

Notably, the increased nitrifiers and denitrifiers and related activities by fertilization suggest the considerable potential for N losses through nitrate leaching and gaseous N (Hu et al., 2015; Tang et al., 2016). For instance, the higher increase of *narG* abundance in the CF + OM soil profile coincided with the substantially higher soil nitrate concentration. Moreover, the ratios of *amoA*/*narG* abundances in subsoil depths were substantially reduced by CF and CF + OM applications (Figure 4). The ratios of (*nirK* + *nirS*)/*nosZ* abundances were increased by the CF and CF + OM treatments in the 20-40 cm but did not change in the 40-80 cm depths, indicating that the ratio of N_2_ to N_2_O as end product of denitrification might be not affected by fertilization in deeper soil depths. These results consistently suggest that loss of N via gaseous emissions in the subsoil profile might be increased by the chronic fertilizer application. This highlights that it is necessary to consider the soil profile to attain a more complete perspective of fertilization effects on N cycle in arable croplands.

## Conclusion

Our results highlight the important effects of the chronic fertilizer applications on the soil profile biogeochemical properties in arable croplands, leading to higher N cycling rates. Denitrifier groups were enriched in the CF subsoil profile, suggesting that loss of N via gaseous emissions in subsoil might be enhanced. Additionally, the abundances of *nirS*- and/or *nirK*-type denitrifiers in subsoil were significantly correlated with DEA. Such changes might contribute to the depleted or less changed total N in the subsoil depths. Overall, our results highlight the necessity to consider the soil profile for fertilization effects on the N cycle, to attain a more complete perspective and a more precise assessment of the effects of fertilization on soil and ecosystem functioning in arable croplands.

## Data Availability Statement

All datasets analyzed for this study are included in the article/Supplementary Material.

## Author Contributions

YW was responsible for the experimental design, data processing, and article writing. YW and HJ collected the samples. HJ and RW contributed to the physiochemical data of soil samples. YH and SG contributed in reviewing the manuscript. All authors contributed to the article and approved the submitted version.

## Conflict of Interest

The authors declare that the research was conducted in the absence of any commercial or financial relationships that could be construed as a potential conflict of interest.
