# Supplementary material for: Synthetic Fertilizer Increases Denitrifier Abundance and Depletes Subsoil Total N in a Long-Term Fertilization Experiment
Source: Front Microbiol. 2020 Aug 31;11:2026. doi: 10.3389/fmicb.2020.02026 (PMC7487435; doi:10.3389/fmicb.2020.02026)
Supplement: Supplementary file 1 [file Data_Sheet_1.pdf]

Supplemental tables and figures

Supplementary Table S1 Spearman correlations of the abundances of nitrifiers and denitrifiers.

Soil depth (cm)		AOA	AOB	nirK	nirS	nosZ
All	AOB	0.656**				
	nirK	0.535**	0.804**			
	nirS	0.505**	0.844**	0.853**		
	nosZ	0.487**	0.831**	0.830**	0.921**	
	narG	0.647**	0.749**	0.585**	0.784**	0.748**
0-20						
	AOB	0.898**				
	nirK	0.644	0.667*			
	nirS	0.915**	0.933**	0.767*		
	nosZ	0.746*	0.783*	0.433	0.683*	
	narG	0.424	0.467	0.000	0.467	0.650
20-40						
	AOB	0.900**				
	nirK	0.750*	0.833**			
	nirS	0.783*	0.833**	0.967**		
	nosZ	0.883**	0.850**	0.867**	0.900**	
	narG	0.983**	0.883**	0.717*	0.767*	0.867**
40-60						
	AOB	0.333				
	nirK	0.583	0.800**			
	nirS	0.267	0.850**	0.917**		
	nosZ	0.433	0.933**	0.817**	0.833**	
	narG	0.400	0.750*	0.833**	0.833**	0.833**
60-80						
	AOB	0.067	1.000			
	nirK	0.167	-0.700*			
	nirS	0.250	0.117	0.133		
	nosZ	0.250	-0.183	0.433	0.867**	
	narG	0.200	0.267	-0.067	0.900**	0.667*

* $P < 0.05$
** $P < 0.01$

Supplementary Table S2 Spearman correlations of soil parameters with the abundances of nitrifiers and denitrifiers.

Soil depth (cm)		DOC	MBN	Nitrate	AP
All	AOA	0.459**	0.357*	0.628**	0.553**
	AOB	0.348*	0.678**	0.714**	0.780**
	AOA/AOB	0.239	0.736**	0.496**	0.681**
	nosZ	0.216	0.777**	0.719**	0.849**
	nirK	0.208	0.687**	0.639**	0.837**
	nirS	0.205	0.714**	0.782**	0.908**
	narG	0.460**	0.466**	0.775**	0.794**
0-20					
	AOA	0.492	0.848**	0.915**	0.915**
	AOB	0.633	0.800**	0.767*	0.867**
	AOA/AOB	0.383	0.217	0.000	0.133
	nosZ	0.583	0.650	0.617	0.783*
	nirK	0.000	0.467	0.550	0.700*
	nirS	0.533	0.817**	0.883**	0.917**
	narG	0.767*	0.400	0.433	0.467
20-40					
	AOA	-0.300	0.767*	0.933**	0.867**
	AOB	-0.150	0.650	0.783*	0.833**
	AOA/AOB	0.483	0.333	0.417	0.517
	nosZ	-0.217	0.733*	0.950**	0.883**
	nirK	0.117	0.667*	0.783*	0.867**
	nirS	0.067	0.667*	0.817**	0.867**
	narG	-0.350	0.733*	0.917**	0.900**
40-60					
	AOA	0.567	-0.417	0.583	0.633
	AOB	0.150	0.200	0.867**	0.817**
	AOA/AOB	0.150	0.350	0.567	0.517
	nosZ	0.300	0.300	0.967**	0.850**
	nirK	0.083	-0.067	0.800**	0.967**
	nirS	0.000	0.133	0.750*	0.883**
	narG	0.067	-0.083	0.733*	0.783*
60-80					
	AOA	-0.167	0.483	0.117	0.050
	AOB	-0.567	-0.100	-0.450	-0.250
	AOA/AOB	-0.550	-0.200	-0.533	-0.450
	nosZ	0.700*	-0.450	0.533	0.650
	nirK	0.467	-0.317	0.150	0.350
	nirS	0.467	-0.383	0.433	0.750*
	narG	0.433	-0.200	0.483	0.750*

* $P < 0.05$
** $P < 0.01$
[a] DOC: dissolved organic C; MBN: microbial biomass N;. AP: available P

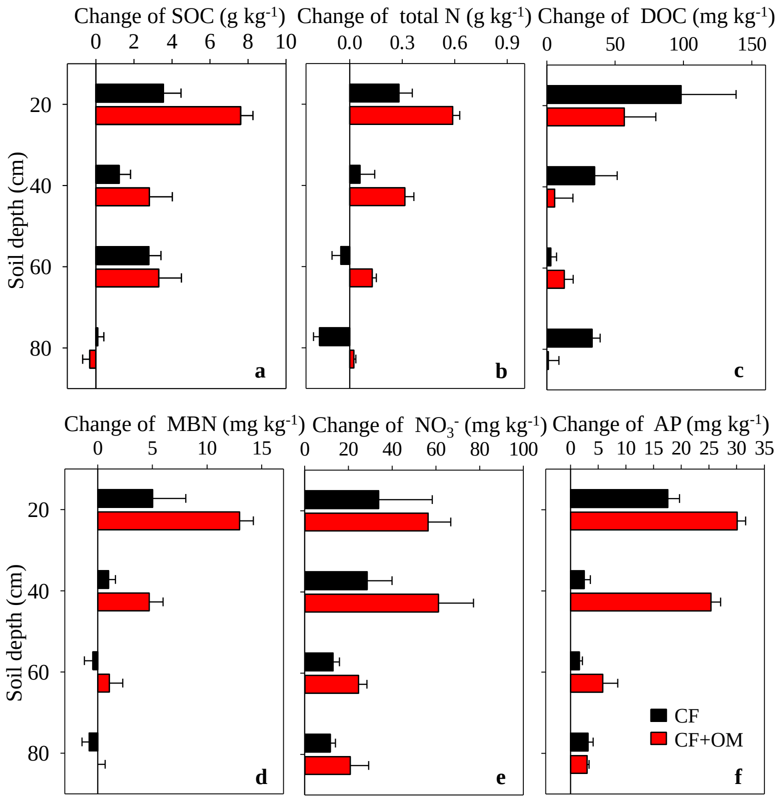

Supplementary Figure S1 Changes in soil parameters in the soil profile (0-80 cm depths) under long-term applications (30 years) of inorganic fertilizer alone (CF) and combined with organic manure (CF+OM) compared with the unfertilized treatment (CK). **a** Soil organic C (SOC); **b** Total N; **c** Dissolved organic C (DOC); **d** Microbial biomass N (MBN); **e** Nitrate; **f** Available P. Error bars are standard errors (*n* = 3).

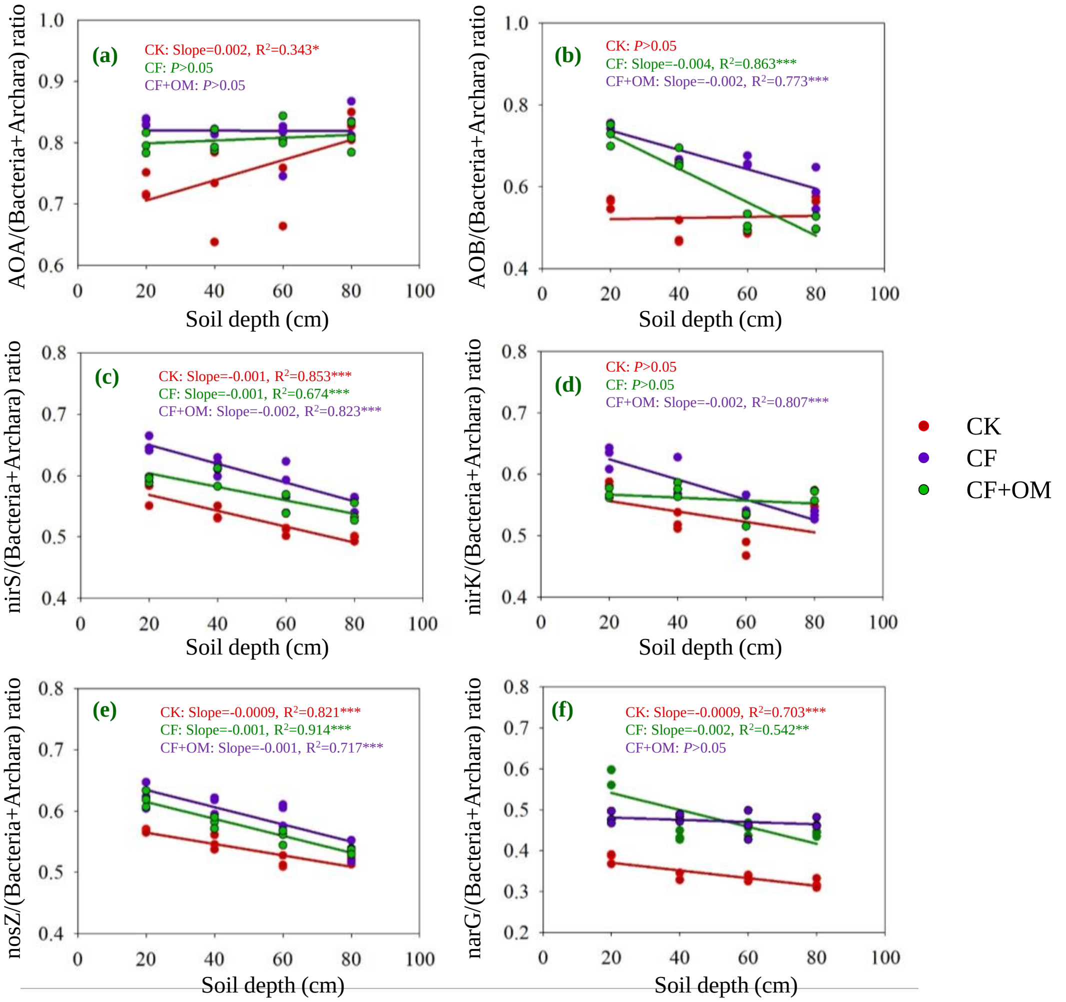

Supplementary Figure S2 The ratios of functional gene/(bacteria+archaea) across soil depths under different fertilizer treatments. The lines denote the least-squares linear regressions across soil depth. * $P < 0.05$; ** $P < 0.01$; *** $P < 0.001$. The ratio of each gene to the sum of bacteria and archaea was calculated as log (functional gene number g^{-1} dry soil)/log (bacterial and archaeal 16S rRNA gene number g^{-1} dry soil). CK: unfertilized soil; CF: inorganic fertilizer; CF+OM: CF plus organic manure.
